# Relative Importance of Mode of Administration in Treatment Preferences among Plaque Psoriasis Patients in the United States

**DOI:** 10.36469/9817

**Published:** 2016-08-10

**Authors:** Steven R. Feldman, Anders Holmen Moeller, Sandra T. Erntoft Idemyr, Juan Marcos González

**Affiliations:** 1 Wake Forest University School of Medicine, Medical Center Boulevard, Winston-Salem, NC, United States; 2 LEO Pharma A/S, Industriparken 55, Ballerup, Denmark; 3 RTI Health Solutions, 200 Park Offices Drive, Research Triangle Park, NC, 27709, United States

**Keywords:** psoriasis, patient preferences, mode of administration, minimum acceptable benefit, discrete-choice experiment

## Abstract

**Background:** Some aspects of psoriasis treatments can negatively influence patients’ quality of life. There is evidence from previous preference-elicitation research in psoriasis that administration characteristics are at least as important as treatment outcomes to patients.

**Objectives:** Our objective was to test the hypothesis that patients’ preferences for reduced disease and treatment burden are as important as preferences around treatment efficacy. We evaluated patient preferences for attributes of psoriasis treatments, including efficacy, tolerability, and mode and frequency of administration.

**Methods:** Adult patients in the United States with a self-reported physician diagnosis of psoriasis completed an online discrete-choice experiment survey. The survey included eight choice questions, each asking respondents to choose between pairs of hypothetical psoriasis medications defined by attributes including efficacy, adverse reactions, and mode and frequency of administration. A random-parameters logit regression model was used to model the preference data. Results from this model were used to calculate respondents’ willingness to trade efficacy for reduced treatment burden.

**Results:** A total of 397 respondents, with a mean self-assessed Psoriasis Activity and Severity Index score of 8.2 (SD, 9.8), provided data for analysis. Improvements in treatment efficacy were more important than improvements in speed of onset and were more important than most increases in the chance of treatment side effects. The maximum possible improvement in treatment efficacy offered in the study was not enough to match the improvements in well being associated with some changes in mode of administration. For example, respondents were willing to accept a reduction in the percentage of patients who achieve clear or almost-clear skin after treatment from approximately 70% to 40% to avoid injections at home and use a topical treatment. Topical treatments were the most preferred option of administration followed by oral agents and intravenous infusion.

**Conclusions:** Psoriasis patients had well-defined preferences for changes in the treatment attributes considered. Avoiding injections in favor of oral or topical treatment was more important to patients than some improvements in efficacy. These findings support previous research regarding the importance of treatment burden relative to outcomes in psoriasis and emphasize the importance of individual patient preferences in determining treatment strategy.

## BACKGROUND

Psoriasis affects patients in many ways and has an enormous impact on patients' quality of life, comparable with that of diabetes or cancer.[1-3] Among the bothersome aspects of psoriasis are the characteristics of treatment.[4-7] Patients' negative opinions of psoriasis treatments relate to the frequency of administration and cosmetic qualities of topical treatments, fear of side effects, and slow onset of action.[6] Patients with psoriasis may find their treatment regimens inconvenient, whether they involve daily home treatment (e.g., with topical or oral agents) or regular clinic visits (e.g., for intravenous infusions with biologic agents).[7] Biologic treatments, although highly effective for patients with moderate to severe psoriasis, carry the risk of potentially severe adverse effects.[5] Disturbing characteristics of treatment reduce patients' quality of life and impede patients' adherence to treatment, a major hurdle limiting treatment effectiveness.[1,8-12]

Previous preference-elicitation work in psoriasis has mostly focused on the evaluation of outcomes or characteristics of current treatments. The impact of the available psoriasis medications on patient wellbeing has been evaluated using health-state utilities3 and the relation of these utilities to patient-reported outcomes.[13] Patient relative preferences for attributes of current treatments have been elicited through choice experiments,[14,15] in particular an evaluation of the benefits versus risks of specific treatment lines, mostly biologics/injectables,[16-18] and an assessment of monetized value (i.e., willingness to pay) for treatment benefits.[19-22]

Some research has specifically evaluated patient preferences for the administration characteristics of psoriasis treatments.[4,23,24] Housman et al., using a validated measure to evaluate vehicle preferences among patients receiving treatment for scalp psoriasis, found that patients preferred less-messy vehicles (i.e., foams and solution preparations) to traditional cream, ointment, and gel vehicles.[4,23] Two unanticipated findings resulted from this evaluation: there were minimal differences in patient preferences between cream and ointment formulations, despite the common conception that patients greatly preferred cream formulations; and there were no differences in preferences between daytime and nighttime administration, despite an expectation that nighttime administration would be preferred.[4,23]

Previous preference research also has found that administration characteristics (e.g., mode, frequency, and location) can be as important as or more important than some treatment outcomes to patients.[24] Schaarschmidt et al. conducted a conjoint-analysis survey to evaluate patients' relative preferences among six outcome-related psoriasis treatment attributes (probability, magnitude, and duration of benefit; probability, reversibility, and severity of adverse events) and five process-related treatment attributes (location, frequency, delivery method, duration, and cost to the patient).[24] The attributes most important to patients were found to be treatment location, probability of benefit, and method of delivery. Nearly all the processrelated attributes had a greater relative importance than the outcome-related attributes; the exceptions were probability of benefit (the second most important attribute overall) and cost to the patient (the least important outcome attribute, less important than both probability and magnitude of benefit).

The goal of this study was to test the hypothesis that patients' preferences for reduced disease burden are as important as preferences around treatment efficacy by formally measuring and testing patients' willingness to trade off clinical benefits for improvements in mode of administration. We present the results of discretechoice experiment (DCE) survey conducted to evaluate patient preferences for the mode of administration of psoriasis treatments.

## METHODS

### Sample

Patients were eligible to participate in the survey if they were aged 18 years or older, were a resident of the United States (US), and had a self-reported physician's diagnosis of psoriasis. An online research panel (All Global) recruited patients with psoriasis (hereafter described as respondents) from existing panels in the US and administered an online survey in July 2014. The study was reviewed by the Office of Research Protection and Ethics at RTI International (the responsible study organization) and approved by its institutional review board. All respondents provided online informed consent.

### Discrete-Choice Experiment

We designed and administered a DCE survey to elicit preferences of patients in the US for attributes of psoriasis treatments, including efficacy, tolerability, and mode and frequency of administration. Discretechoice experiments offer a systematic method of eliciting tradeoffs to quantify the relative importance that health care decision makers place on various treatment characteristics or outcomes.[25-28] Discrete-choice experiments are based on the premise that treatments are bundles of attributes or features (e.g., efficacy and side effects) and that the value given to treatments can be decomposed into the relative value that each treatment attribute has to individuals. Thus, choices between treatments also reflect the relative value of the treatment attributes.[29] Systematic analysis of treatment choices under experimental control can produce attribute-specific weights that indicate the importance of each attribute in respondents' choices.[29,30] Results from DCE surveys can be used to establish patients' willingness to accept reductions in treatment efficacy to avoid the burden of treatment administration. This comparison could further demonstrate the degree to which patients see value in reducing the burden of treatment administration.

### Survey Instruments

A DCE survey was developed and administered online to respondents in the US following the good practice recommendations issued by the International Society for Pharmacoeconomics and Outcomes Research Conjoint Analysis Task Force.27 The survey was divided into four components: background questions (regarding psoriasis severity, psoriasis history, and treatment experience); attribute definitions and comprehension questions; preference-elicitation questions; and demographic characteristics.

### Choice Questions

The DCE survey included a series of choice questions asking respondents to choose between pairs of hypothetical psoriasis medications defined by a combination of attributes of these therapies (Table 1). A preliminary list of attribute levels and medication features to include in the survey instrument was developed based on feedback from participants in two focus groups conducted in Philadelphia, Pennsylvania, with a total of 18 adults with plaque psoriasis. The focus groups were conducted to elicit information on participants' experiences with different types of psoriasis treatments, the salience of different medication features, participants' satisfaction with current treatments, and participants' perceptions of the unmet needs with current treatments.

The appropriateness of the attributes and attribute levels included in the final choice questions was confirmed through in-person semistructured pretest interviews conducted in Raleigh, North Carolina, with 15 individual regional respondents. Respondents were invited to participate in the pretest interviews if they reported having a physician's diagnosis of psoriasis. The interviews were designed to test the clarity, appropriateness, and difficulty of the survey content.

**Table 1.** Attributes and Levels Included in the Survey Instrument

| Attribute | Level |
|---|---|
| Patients who had clear or almost-clear skin after taking the medicine | 80 out of 100 (80%) |
| | 60 out of 100 (60%) |
| | 40 out of 100 (40%) |
| Time until patients had clear or almost-clear skin if the medicine worked | One week |
| | Two weeks |
| | One month |
| | Two months |
| How you take the medicine | Topical |
| | Oral |
| | Injection at home |
| | Injection at doctor's office |
| | Intravenous infusion (IV) |
| How often you need to take the medicine | Once a month |
| | Once a week |
| | 3 times a week |
| | Once a day |
| | Twice a day |
| Skin reactions or stomach problems | None |
| | Moderate stomach problems |
| | Moderate skin reaction |
| Chance of severe headaches during the first few weeks of treatment | 0 out of 100 (0%) |
| | 5 out of 100 (5%) |
| | 20 out of 100 (20%) |
| Chance of getting pneumonia during the first year of treatment | 0 out of 100 (0%) |
| | 1 out of 100 (1%) |
| | 2 out of 100 (2%) |
| Personal cost to you each month | $20 |
| | $75 |
| | $150 |

After pretesting the survey instrument, an experimental design was created to populate the hypothetical treatments presented to respondents in the choice questions. The experimental design ensured enough independent variation in the levels of hypothetical profiles to identify preferences for each attribute level through respondents' treatment choices. Good research practices were used to construct a fractional factorial experimental design.[30-32] Respondents were randomly assigned to one of five versions of the survey, each with eight choice questions from the experimental design (Figure 1).

**Figure 1.** Example of the Treatment-Choice Question

| Medicine Feature | Medicine A | Medicine B |
|---|---|---|
| Patients who had clear or almost clear skin after taking medicine | 80 out of 100 (80%) | 60 out of 100 (60%) |
| Time until patients had clear or almost clear skin if the medicine worked | Two weeks | One month |
| How you take the medicine | Intravenous Infusion (IV) | Topical |
| How often you need to take the medicine | Once a week | Once a day |
| Skin reactions or stomach problems | Moderate skin reaction | Moderate stomach problems |
| Chance of severe headaches during the first few weeks of treatment | 0 out of 100 (0%) | 5 out of 100 (5%) |
| Chance of getting pneumonia during the first year of treatment | 1% (1 out of 100) | 0 out of 100 (0%) |
| Personal cost to you each month | $150 | $20 |
| **Which medicine would you choose?** | ☐ | ☐ |

## Study Analysis

*Summary of Disease Severity*

Based on each respondent's self-reported lesion severity and coverage, we calculated a Self Assessed Psoriasis Area and Severity Index (SAPASI) score to assess disease severity, as follows:

- Step 1: For each of the four body areas (head, upper extremities, trunk, lower extremities), respondents provided a score of 0 to 4 (none, slight, mild, moderate, severe) to indicate the severity of erythema (redness), induration (thickness), and desquamation (scales). Respondents also indicated the percentage of each body area affected by psoriasis (i.e., involvement).

- Step 2: We summed the three severity scores for each of the four body areas. The total score for each body area ranged from 0 to 12.
- Step 3: We multiplied the total score for each body area by the score for the percentage involvement in that body area. The involvement scores were 1 if involvement was 10% or less, 2 if involvement was 11% to 30%, 3 if involvement was 31% to 50%, 4 if involvement was 51% to 70%, 5 if involvement was 71% to 90%, and 6 if involvement was 91% to 100%.
- Step 4: We multiplied the scores from step 3 by 0.1 if lesions were reported on the head, 0.2 if reported on the upper extremities, 0.3 if reported on the trunk, and 0.4 if reported on lower extremities.
- Step 5: We summed the scores from step 4 for each respondent. The maximum possible SAPASI score is 72.

*Analysis of Choice Questions*

A random-parameters logit (RPL) regression model was used to analyze the preference data from the survey, specifically by estimating the relative preference weights for each attribute level in the study based on respondents' answers to choice questions.[33] Random-parameters logit relates the probability of choosing one treatment over another to the observable differences between the treatment options. In that sense, the model estimated the relative impact of changes in treatment attributes on the probability of choice and relied on that information to determine the relative marginal benefit/harm of the changes. The marginal relative benefit/harm of attribute changes in an RPL is known as log-odds or preference weights for an attribute level. For example, increasing the percentage of patients who had clear or almost-clear skin after taking a medicine increased patients' stated preference for psoriasis treatments and the weight that respondents gave that attribute in favor of a psoriasis treatment. The RPL regression model considered variations in preferences across respondents and approximated the distribution of these preferences by estimating a standard deviation for each attribute level. This estimate indicated how much the weights for an attribute level varied across the respondents in the sample.

Preferences for all attributes were estimated assuming a categorical representation of the attribute levels. The categories for each attribute were effects-coded (instead of dummy-coded), which allowed for the estimation of a preference weight for all attribute levels. This implies that the preference weight for one level in each attribute was constrained to be the negative sum of all other levels in the attribute for identification purposes. Mean preference weights from the RPL model indicated the average difference between the preference weight for each attribute level and the mean effect for the attribute. Positive estimates for preference weights indicate that the level increased the chance of choosing a medication relative to the mean attribute impact. Negative estimates for preference weights indicate that the level decreased the chance of choosing a medication relative to the mean attribute impact. The standard deviation parameters from the RPL model indicate the dispersion of the distribution of preferences across respondents. Greater estimates for the standard deviation parameters indicate greater variation in preferences for an attribute level as detected in the pattern of choices from each respondent.

*Calculating Minimum Acceptable Benefit*

Minimum acceptable benefit is the minimum change in treatment efficacy respondents would require to accept a specific change in other aspects of treatment. The minimum acceptable benefit was calculated as the change in the percentage of patients who would have clear or almost-clear skin after taking a psoriasis medication

(i.e., the chance that a patient taking the medication would have clear or almost-clear skin) that would be as important to patients as a specific modification in the way psoriasis medications are administered (i.e., mode of administration). This equivalence was established using the preference weights for treatment efficacy and mode of administration obtained through the RPL regression model. Starting from a baseline efficacy level of a 40% chance of achieving clear or almost-clear skin, the change in efficacy that offset increased treatment burden due to modifications in mode of administration was identified as the minimum acceptable benefit.

## RESULTS

### Response Rate

A total of 1,197 people from existing panels in the US expected to meet the study inclusion criteria were invited to be screened for eligibility. Of those invited, 579 individuals responded to the invitation. Of those who responded, 458 (79%) were eligible to participate because they self-reported a physician diagnosis of psoriasis. Of those who were eligible to participate, 404 (88%) consented to participate and completed the survey. A total of 7 respondents always chose the same answer, Medicine A or Medicine B. These respondents were excluded from the final sample and subsequent analysis as lack of response variation in the choice questions is considered an indication that respondents did not pay close attention to the questions in the survey. The final study sample was 397 (= 404 – 7) respondents.

### Sample Characteristics

Table 2 presents the characteristics of the final sample of respondents (N = 397). The mean (standard deviation) age was 49 (16) years, and approximately two-thirds of the sample was younger than 58 years. Sixty-one percent of the respondents were female. More than one third of respondents (35%) had received a psoriasis diagnosis at least 10 years before the survey. A total of 383 respondents (97%) were taking a topical treatment, 62 (16%) were taking a biologic, and 82 (21%) were taking an oral treatment (some respondents were taking multiple treatments); 91% reported using prescription or over-the-counter creams, lotions, and ointments. Most respondents reported having lesions on their head (63.2% of respondents) and arms (64.5% of respondents). Table 3 presents the respondents' reported psoriasis severity by body area.

**Table 2.** Patient Sample Characteristics (N = 397)

| Characteristic | Statistic or Category | Overall (N = 397) |
|---|---|---|
| **All respondents** | | |
| Sex | n | 397 |
| | Female | 242 (61.0%) |
| | Male | 155 (39.0%) |
| Age (years) | n | 397 |
| | Mean (SD) | 48.8 (15.5) |
| | Median | 50.0 |
| | Minimum, maximum | 19.0, 84.0 |
| Have you ever been diagnosed with any of the following medical conditions? (Check all thatapply.)[a] | n | 397 |
| | High blood pressure | 136 (34.3%) |
| | Arthritis | 129 (32.5%) |
| | High cholesterol | 125 (31.5%) |
| | Asthma or allergies | 98 (24.7%) |
| | Migraines | 85 (21.4%) |
| Which of the following treatments do you currently use to treat your psoriasis? (Please check all that apply.)[a] | n | 397 |
| | Creams, lotions, and ointments (includes prescription and over-the-counter) | 362 (91.2%) |
| | Shampoos | 165 (41.6%) |
| | Prescription oral medicines | 82 (20.7%) |
| | Light therapy | 42 (10.6%) |
| | Injectable medicines | 52 (13.1%) |
| | Intravenous infusions at a doctor's office, infusion center, or hospital | 19 (4.8%) |
| Where do you currently have active psoriasis patches? (Please check all that apply.)[a] | n | 397 |
| | Head (including face, hair, scalp, and neck) | 251 (63.2%) |
| | Arms (including elbows, wrists, and hands) | 256 (64.5%) |
| | Legs (including hips, knees, ankles, and feet) | 217 (54.7%) |
| | Torso (including chest, stomach, back, genital area, and buttocks) | 140 (35.3%) |
| | I don't currently have any psoriasis patches | 8 (2.0%) |
| About how long ago were you diagnosed with psoriasis? | n | 397 |
| | 2 years or more | 310 (78.1%) |
| Current treatment for psoriasis[a] | n | 397 |
| | Topical | 383 (96.5%) |
| | Oral | 82 (20.7%) |
| | Systemic | 62 (15.6%) |
| SAPASI score | n | 392 |
| | Mean score (SD) | 8.2 (9.8) |
| | Missing | 5 |
| Disease severity score (based on SAPASI score) | n | 392 |
| | > 12 (severe level) | 90 (23.0%) |
| | ≤ 12 (mild level) | 302 (77.0%) |
| | Missing | 5 |

SAPASI = Self Assessed Psoriasis Area and Severity Index; SD = standard deviation.
Note: Percentages do not include missing responses in the denominator.
[a]Respondents were allowed to select more than one answer to this question. Thus, the sum of responses across categories does not necessarily add up to the number of respondents who answered the question.

**Table 3.** Respondents' Reported Psoriasis Severity, by Body Area (N = 392)

| | Head (n = 251) | Arms (n = 256) | Legs (n = 217) | Torso (n = 140) |
|---|---|---|---|---|
| Redness: 0-4, mean (SD) | 2.2 (1.0) | 2.2 (1.0) | 2.4 (1.1) | 2.5 (1.0) |
| Thickness: 0-4, mean (SD) | 2.1 (1.0) | 2.0 (0.9) | 2.2 (1.0) | 2.1 (1.0) |
| Scaling: 0-4, mean (SD) | 2.4 (1.0) | 2.2 (1.0) | 2.4 (1.0) | 2.1 (1.0) |
| ≥ 30% covered with psoriasis patches | 78 (31.1%) | 72 (28.1%) | 74 (34.1%) | 37 (26.4%) |

SD = standard deviation.

## Patient Preferences Among Treatment Attributes

The RPL model results are divided into two groups of parameters, mean preference weights and standard deviation parameters (Table 4). Both groups of parameters include standard errors. The standard errors in the regression model result characterize the uncertainty around the mean preference weights and the variation of these preference weights across respondents (standard deviations).

**Table 4.** Mean Preference Estimates and Standard Deviation of Preference Estimates Based on Treatment Choices for the Pooled Sample (N = 397)

| Attribute | Level | Coefficient | Standard Error | Test-Statistic | P Value[a] |
|---|---|---|---|---|---|
| **Mean preference estimates** | | | | | |
| Patients who had clear or almost-clear skin after taking the medicine | 80 out of 100 (80%) | 0.653 | 0.077 | 8.490 | 0.000 |
| | 60 out of 100 (60%) | 0.018 | 0.050 | 0.370 | 0.711 |
| | 40 out of 100 (40%) | –0.672 | 0.080 | –8.364 | 0.000 |
| Time until patients had clear or almost-clear skin if the medicine worked | One week | 0.254 | 0.078 | 3.270 | 0.001 |
| | Two weeks | 0.074 | 0.073 | 1.010 | 0.312 |
| | One month | –0.069 | 0.066 | –1.040 | 0.299 |
| | Two months | –0.258 | 0.075 | –3.456 | 0.001 |
| How you take the medicine | Topical | 0.921 | 0.098 | 9.380 | 0.000 |
| | Oral | 0.562 | 0.105 | 5.370 | 0.000 |
| | Injection at home | –0.102 | 0.092 | –1.110 | 0.268 |
| | Injection at doctor's office | –0.291 | 0.091 | –3.200 | 0.001 |
| | Intravenous infusion (IV) | –1.091 | 0.134 | –8.162 | 0.000 |
| How often you need to take the medicine | Once a month | 0.430 | 0.084 | 5.115 | 0.000 |
| | Once a week | –0.021 | 0.093 | –0.220 | 0.826 |
| | 3 times a week | –0.292 | 0.089 | –3.280 | 0.001 |
| | Once a day | –0.026 | 0.101 | –0.260 | 0.794 |
| | Twice a day | –0.091 | 0.096 | –0.940 | 0.346 |
| Skin reactions or stomach problems | None | 0.296 | 0.058 | 5.110 | 0.000 |
| | Moderate skin reaction | 0.021 | 0.062 | 0.337 | 0.736 |
| | Moderate stomach problems | –0.317 | 0.065 | –4.890 | 0.000 |
| Chance of severe headaches during the first few weeks of treatment | 0 out of 100 (0%) | 0.440 | 0.070 | 6.320 | 0.000 |
| | 5 out of 100 (5%) | 0.144 | 0.054 | 2.660 | 0.008 |
| | 20 out of 100 (20%) | –0.584 | 0.072 | –8.134 | 0.000 |

**Table 4.** Mean Preference Estimates and Standard Deviation of Preference Estimates Based on Treatment Choices for the Pooled Sample (N = 397) - continued

| Attribute | Level | Coefficient | Standard Error | Test-Statistic | P Value[a] |
|---|---|---|---|---|---|
| **Mean preference estimates - continued** | | | | | |
| Chance of getting pneumonia during the first year of treatment | 0 out of 100 (0%) | 0.203 | 0.066 | 3.090 | 0.002 |
| | 1 out of 100 (1%) | 0.010 | 0.056 | 0.170 | 0.863 |
| | 2 out of 100 (2%) | –0.213 | 0.065 | –3.292 | 0.001 |
| Personal cost to you each month | $20 | 0.812 | 0.085 | 9.570 | 0.000 |
| | $75 | 0.021 | 0.059 | 0.350 | 0.724 |
| | $150 | –0.833 | 0.081 | –10.229 | 0.000 |
| **Standard deviation estimates** | | | | | |
| Patients who had clear or almost-clear skin after taking the medicine | 80 out of 100 (80%) | 0.720 | 0.093 | 7.710 | 0.000 |
| | 60 out of 100 (60%) | 0.077 | 0.116 | 0.670 | 0.503 |
| | 40 out of 100 (40%) | 0.797 | 0.155 | 5.160 | 0.000 |
| Time until patients had clear or almost-clear skin if the medicine worked | One week | 0.193 | 0.150 | 1.290 | 0.199 |
| | Two weeks | 0.133 | 0.181 | 0.730 | 0.464 |
| | One month | 0.271 | 0.138 | 1.970 | 0.049 |
| | Two months | 0.596 | 0.278 | 2.148 | 0.032 |
| How you take the medicine | Topical | 0.907 | 0.123 | 7.370 | 0.000 |
| | Oral | 0.416 | 0.272 | 1.530 | 0.126 |
| | Injection at home | 0.150 | 0.248 | 0.610 | 0.545 |
| | Injection at doctor's office | 0.027 | 0.120 | 0.220 | 0.825 |
| | Intravenous infusion (IV) | 1.500 | 0.359 | 4.178 | 0.000 |
| How often you need to take the medicine | Once a month | 0.382 | 0.259 | 1.477 | 0.140 |
| | Once a week | 0.080 | 0.111 | 0.720 | 0.471 |
| | 3 times a week | 0.040 | 0.116 | 0.340 | 0.732 |
| | Once a day | 0.067 | 0.134 | 0.500 | 0.618 |
| | Twice a day | 0.195 | 0.166 | 1.170 | 0.241 |
| Skin reactions or stomach problems | None | 0.221 | 0.110 | 2.010 | 0.045 |
| | Moderate skin reaction | 0.309 | 0.188 | 1.648 | 0.099 |
| | Moderate stomach problems | 0.088 | 0.129 | 0.680 | 0.495 |
| Chance of severe headaches during the first few weeks of treatment | 0 out of 100 (0%) | 0.713 | 0.087 | 8.160 | 0.000 |
| | 5 out of 100 (5%) | 0.076 | 0.104 | 0.730 | 0.465 |
| | 20 out of 100 (20%) | 0.789 | 0.141 | 5.596 | 0.000 |
| Chance of getting pneumonia during the first year of treatment | 0 out of 100 (0%) | 0.240 | 0.096 | 2.510 | 0.012 |
| | 1 out of 100 (1%) | 0.044 | 0.119 | 0.370 | 0.714 |
| | 2 out of 100 (2%) | 0.284 | 0.158 | 1.798 | 0.072 |
| Personal cost to you each month | $20 | 0.971 | 0.105 | 9.270 | 0.000 |
| | $75 | 0.163 | 0.074 | 2.210 | 0.027 |
| | $150 | 1.134 | 0.110 | 10.344 | 0.000 |

[a]The P value measures the probability that the coefficient is different from zero, where zero is the mean effect.

Note: Mean preference estimates are log-odds coefficients representing the relative benefit/harm of an attribute level relative to the mean effect for that attribute, which was normalized to zero. The standard deviation estimates characterize the expected variation in mean preference estimates across respondents in the sample. Both types of estimates are based on respondents' choices in the DCE questions.

To clarify the preference information characterized by the RPL model results, Figure 2 displays the mean preference weights (and 95% confidence interval) for the respondents. The vertical distance between preference weights represents the relative importance of moving from one level of an attribute to another level of the same attribute measured in log-odds.

**Figure 2.** Patient Preference Weights (N = 397)

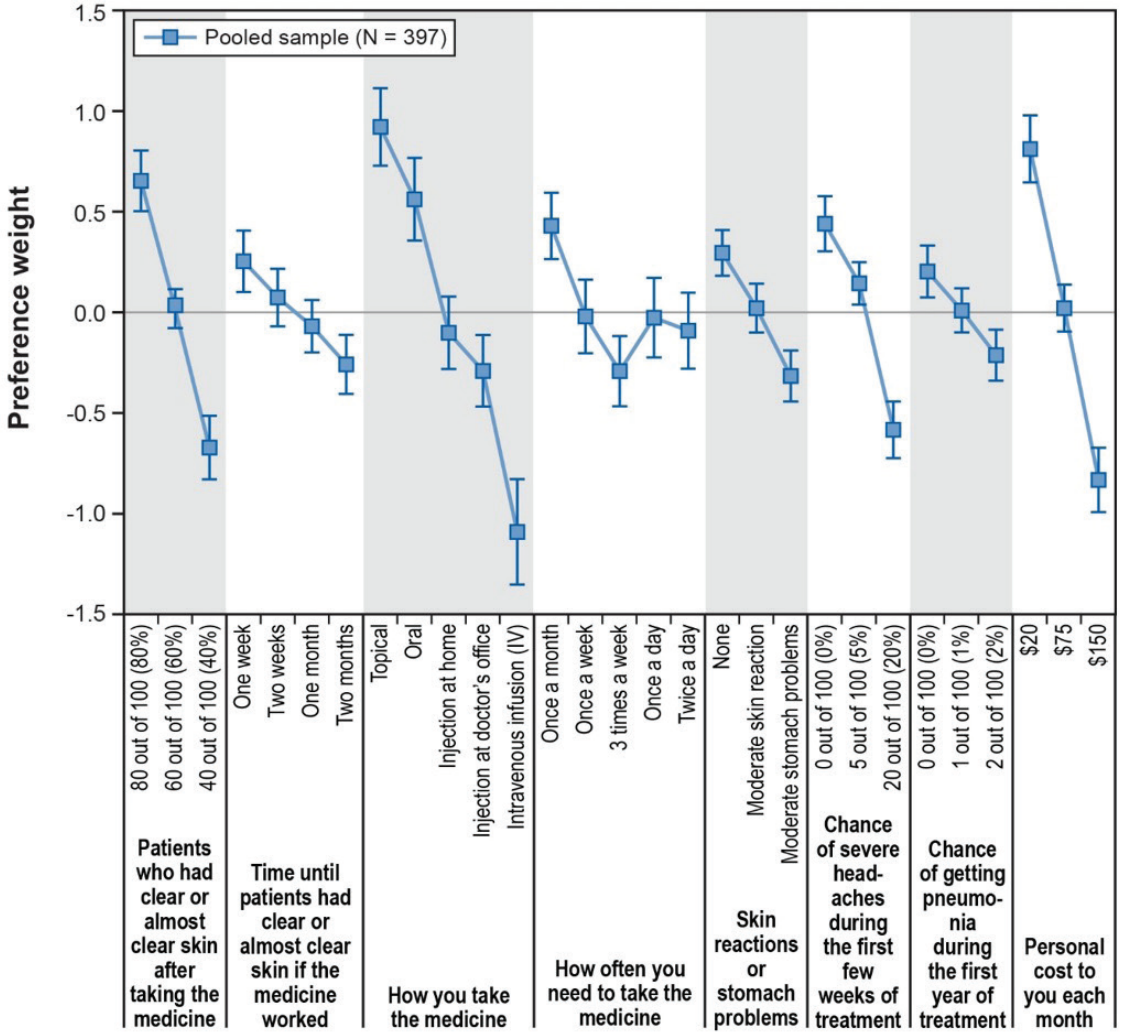

Note: Figure presents the mean preference weights (and 95% confidence interval) for the respondents. The vertical distance between preference weights represents the relative importance of moving from one level of an attribute to another level of the same attribute measured in log-odds.

There was considerable variation in preference across the levels of each attribute in the study. For all attributes, the most and least preferred levels were statistically significantly different at the 95% confidence level. Variation in mean preferences was greatest across options of administration: topical administration was the most preferred option, and infusion was the least preferred option. The maximum improvement in treatment efficacy was as important as the maximum increase in the personal cost for acquiring the medication and more important than all improvements in speed of onset of the medication. Maximum efficacy improvements were also more important than all increases in the chance of treatment-related skin reactions and pneumonia.

Statistical significance of the standard deviation estimates in Table 4 can be associated with significant heterogeneity in the preference weights for attribute levels across respondents. Statistically significant standard deviations were identified in at least some levels of all attributes except for frequency of treatment, suggesting that systematic variations in patients' preferences are pervasive in our data.

We evaluated differences in preferences based on the SAPASI reported by respondents. For two attributes, preferences of respondents with more severe psoriasis (SAPASI>12) were statistically significantly different from preferences of respondents with milder psoriasis (SAPASI≤12, P = 0.03). Unlike respondents with more severe psoriasis, respondents with milder psoriasis did not distinguish between levels of onset of action or between injections and IV infusions at the doctor's office.

### Patients' Willingness to Accept Tradeoffs in Efficacy and Mode of Administration

The maximum possible improvement in treatment efficacy offered in the study was not enough to match the improvements in well-being associated with the avoidance of intravenous infusion when the treatment could be administered topically or orally (Table 5). Patients in our sample were willing to accept on average an injectable over a topical treatment if the efficacy increased by 30 percentage points. Similarly, patients in our sample would accept on average an injectable over an oral treatment if the efficacy increased by 20 percentage points.

**Table 5.** Minimum Acceptable Benefit Estimates, Means, and 95% Confidence Intervals (N = 397)

| Attribute | Change in Administration Mode | Mean MAB of Percentage-Point Improvement in Patients Who Had Clear or Almost-Clear Skin After Taking the Medicine[a] (95% CI) |
|---|---|---|
| Mode of administration (How you take the medicine) | Injection at home to injection at doctor's office | 5.5% (–2.0% to 14.1%) |
| | Topical to oral | 10.4% (3.0%-20.7%) |
| | Oral to injection at home | 19.2% (10.7%-30.5%) |
| | Injection at doctor's office to intravenous infusion (IV) | 23.5% (12.5%-35.5%) |
| | Oral to injection at doctor's office | 25.1% (15.0%-37.9%) |
| | Injection at home to intravenous infusion (IV) | 29.4% (17.7%-43.6%) |
| | Topical to injection at home | 30.5% (20.3%-43.0%) |
| | Topical to injection at doctor's office | 36.4% (24.9%-50.6%) |
| | Oral to intravenous infusion (IV) | 50.3% (36.5%-67.8%)[b] |
| | Topical to intravenous infusion (IV) | 61.6% (46.6%-85.2%)[b,c] |

CI = confidence interval; MAB = minimum acceptable benefit.
[a]From a baseline efficacy level of 40%.
[b]After considering the baseline efficacy level, the mean MAB estimate is above the maximum efficacy level included in the study (80%) and required extrapolating respondents' preferences.
[c]An MAB ≥ 60% implies certain improvement in skin clearance with the medication, as 60% (MAB) + 40% (baseline) = 100%

The minimum acceptable benefits associated with changes in mode of administration are also an indication of the amount of efficacy respondents were willing to forgo under specific circumstances as administration became less burdensome. For example, respondents were willing to accept a 10-percentage-point reduction in the percentage of patients who get clear or almost-clear skin after treatment (from approximately 50% to 40%) to avoid an oral treatment and use a topical treatment. Also, respondents were willing to accept a 30-percentage-point reduction in the percentage of patients who get clear or almost-clear skin after treatment (from approximately 70% to 40%) to avoid injections at home and use a topical treatment.

## DISCUSSION

Our sample shows high participation of women and relatively young respondents (aged < 58 years). Some evidence suggests that the overall psoriasis population in the US is similar in age to the study sample.[34] The sample included more women than men, despite no clear evidence of gender differences in psoriasis.[35] A relatively high proportion of respondents with a SAPASI score of ≤ 12 may suggest that respondents generally had milder psoriasis symptoms. Among the general psoriasis population, approximately 80% of patients have mild to moderate disease, and approximately 20% have moderate to severe disease[36]; thus, the severity distribution of the study population was comparable to that of general psoriasis population. Most respondents had experience with topical treatments, followed by oral treatments and biologic treatments.

Respondents with psoriasis had clear preferences for changes in some attributes as compared with others considered in the study, as well as statistically significant differences in preferences among the attribute levels considered in the study. As found in previous studies,[4,23,24] administration factors were of great importance to respondents. Our findings support those of Schaarschmidt et al.,[24] that some aspects of administration were more important to patients than other clinical factors. Schaarschmidt and colleagues' study was conducted in an outpatient dermatology clinic in Germany, and participants in their study assigned more importance to most process attributes (with the exception of cost, the least important outcome attribute) than to most treatment attributes (with the exception of probability of benefit, the second most important attribute overall).

Our study found that some changes in mode of administration were more important to patients than improvements in efficacy. Respondents were willing to forgo some chance of efficacy to reduce the burden of treatment by avoiding an injectable treatment in favor of a topical or oral treatment. Respondents in our sample were generally averse to injectables and infusions and preferred topical or oral therapies. We also observed statistically significant differences between the burden of topical and oral therapies, with the former being preferred. Respondents generally preferred less frequent application, as long as it was regular (once a month, once a week, once a day, or twice a day). Patients were also sensitive to efficacy and onset of action. Finally, preference heterogeneity was identified for levels in most attributes. Although identifying the source of preference heterogeneity is beyond the objective of this study, this result suggests that preferences for psoriasis treatments can be expected to vary across patients.

These findings have important clinical implications for development of psoriasis treatment guidelines and for individualized psoriasis treatment planning. Other research has revealed patient preferences that contradict aspects of the previously prevailing treatment paradigm in psoriasis, such as Housman and colleagues' findings regarding vehicle preferences among patients with psoriasis[4,23] and Schaarschmidt and colleagues' findings regarding the importance of process-related attributes relative to outcome-related attributes.[24] The results of our study provide evidence that, even after accounting for some differences in treatment efficacy, patients have statistically and clinically meaningful preferences for different modes of administration. These findings complicate the attempts by payer, patient, and professional organizations to create guidelines on how psoriasis should be treated. Guidelines based on efficacy and safety alone may overlook other considerations that are important to patients. Guidelines that provide flexibility for physicians and patients to choose among various reasonable therapeutic options may be appropriate. Moreover, these findings also highlight the difficulty physicians may face in recommending an "ideal" treatment plan to patients. Although physicians may have more information than patients on the efficacy, safety, and other characteristics of potential treatments, patients have more information than physicians on their preferences among those characteristics. This suggests that, in the ideal treatment for an individual patient, the physician and the patient have shared responsibilities and should discuss efficacy, safety, mode of administration, and other relevant clinical aspects of treatment.

### Limitations

This choice-format conjoint-analysis survey asked respondents to evaluate hypothetical treatments for psoriasis. Potential hypothetical bias was minimized by offering alternatives that mimic real-world tradeoffs as closely as possible. However, not all attributes of treatments (e.g., costs, other adverse events, other benefits) were included in the survey, and differences can arise between stated and actual choices. The results from this study relate only to the attributes and attribute levels defined in the survey. Alternative definitions of the attributes and attribute levels could affect the conclusions reached here. The sampling process was not designed or weighted to ensure representativeness of the population of individuals with psoriasis. Moreover, the data represent the average preferences across the group, and specific preferences of individual patients must be considered when making individual clinical decisions.

## CONCLUSIONS

Patients with psoriasis had clear preferences for changes with regard to mode of administration of psoriasis medications given the treatment characteristics included in the study. Avoiding injections in favor of oral or topical treatment was more important than certain improvements in treatment efficacy. In addition, patients clearly preferred medications that were associated with less personal costs and were considered safe; patients also were willing to trade off efficacy in favor of the latter attributes. These findings support observations from previous research regarding the importance of treatment burden relative to treatment outcomes in psoriasis and emphasize the importance of individual patient preferences in determining treatment strategy.

## ACKNOWLEDGMENTS

The authors express their gratitude to Joshua Posner of RTI Health Solutions for his contributions throughout the study. Kate Lothman of RTI Health Solutions provided medical-writing assistance.

### Conflict(s) of Interest

Dr. Feldman received research, speaking, and/or consulting support from a variety of companies, including Galderma, GSK/Stiefel, Leo Pharma, Baxter, Boehringer Ingelheim, Mylan, Celgene, Pfizer, Valeant, AbbVie, Cosmederm, Anacor, Astellas, Janssen, Lilly, Merck, Merz, Novartis, Qurient, National Biological Corporation, Caremark, Advance Medical, Suncare Research, Informa, UpToDate, and National Psoriasis Foundation. He is founder and majority owner of www.DrScore.com, a patient satisfaction survey service, and is a founder and part owner of Causa Research, a company working to enhance patients' adherence to treatment. Anders Holmen Moeller and Sandra T. Erntoft Idemyr are employees of LEO Pharma A/S, the sponsor of this research.
